# Supplementary material for: Safety and Diagnostic Accuracy of the Transnasal Approach for Endobronchial Ultrasound-Guided Transbronchial Needle Aspiration (EBUS-TBNA)
Source: Diagnostics (Basel). 2023 Apr 13;13(8):1405. doi: 10.3390/diagnostics13081405 (PMC10137160; doi:10.3390/diagnostics13081405)
Supplement: Supplementary file 1 [file diagnostics-13-01405-s001.zip › diagnostics-2287170-supplementary.docx]

Table S1. Anagraphics, data regarding samples, procedural sedation, adverse events and results of the whole population and the subgroups. * Mean, SD and p values of propofol and remifentanil have been calculated taking into account only cases with anesthesiologist assistance. SD, standard deviation; BMI, body mass index; NSCLC, non-small cell lung cancer; SCLC, small cell lung cancer.

|  | **Overall population (464)** | **Nose (244)** | **Mouth (173)** | **Laryngeal mask (47)** | **p value** |
| --- | --- | --- | --- | --- | --- |
| **Age (years, mean ± SD)** | 66.7 (± 11.4) | 69.1 (± 11.5) | 68.3 (± 11.4) | 68.1 (± 11.9) | 0.7 |
| **Sex (males, %)** | 296 (63.8) | 169 (69.3) | 96 (55.5) | 31 (66) | 0.01 |
| **Death (%)** | 236 (50.9) | 123 (50.4) | 94 (54.3) | 19 (40.4) | 0.2 |
| **Weight (kg, mean ± SD)** | 73.5 (±16.3) | 74.8 (± 16.4) | 71.7 (± 16.8) | 73.3 (± 12.9) | 0.09 |
| **Height (cm, mean ± SD)** | 167 (± 9.9) | 168.1 (± 10.3) | 165.4 (± 9.2) | 167.3 (± 9.1) | 0.02 |
| **BMI (kg/m^2^, mean ± SD)** | 26.2 (± 4.9) | 26.3 (± 4.6) | 26 (± 5.4) | 26 (± 4.5) | 0.5 |
| **Previous biopsies (%)** | 81 (17.5) | 37 (15.2) | 29 (16.8) | 15 (31.9) | 0.02 |
| **Mediastinal staging (%)** | 136 (29.3) | 74 (30.3) | 37 (21.4) | 25 (53.2) | 0.0001 |
| **Mediastinal adenopathies (%)** | 444 (95.7) | 231 (94.7) | 168 (97.1) | 45 (95.7) | 0.5 |
| **Adequate visualization (%)** | 455 (98.1) | 238 (98.3) | 170 (97.5) | 47 (100) | 0.5 |
| **Sample acquisition (%)** | 458 (98.7) | 240 (98.4) | 171 (98.8) | 47 (100) | 0.7 |
| **N. of stations sampled (mean ± SD)** | 1.7 (± 1) | 1.7 (± 1.1) | 1.6 (± 0.9) | 2.1 (± 1.3) | 0.02 |
| **2R (%)** | 9 (1.9) | 7 (2.9) | 0 (0) | 2 (4.2) | 0.053 |
| **2L (%)** | 3 (0.6) | 3 (1.2) | 0 (0) | 0 (0) | 0.3 |
| **3 (%)** | 3 (0.6) | 2 (0.8) | 0 (0) | 1 (2.1) | 0.2 |
| **4R (%)** | 173 (37.3) | 93 (38.1) | 62 (35.8) | 18 (38.3) | 0.9 |
| **4L (%)** | 64 (13.8) | 34 (13.9) | 16 (9.2) | 14 (29.8) | 0.001 |
| **7 (%)** | 273 (58.8) | 138 (56.6) | 103 (59.5) | 32 (68.1) | 0.4 |
| **8 (%)** | 3 (0.6) | 2 (0.8) | 1 (0.6) | 0 (0) | 0.8 |
| **10R (%)** | 23 (4.9) | 7 (2.9) | 9 (5.2) | 7 (14.9) | 0.002 |
| **10L (%)** | 14 (3) | 8 (3.3) | 5 (2.9) | 1 (2.1) | 0.9 |
| **11R (%)** | 108 (23.3) | 57 (23.4) | 36 (20.8) | 15 (31.9) | 0.8 |
| **11L (%)** | 81 (17.5) | 40 (16.4) | 23 (19.1) | 8 (17) | 0.3 |
| **12R (%)** | 0 (0) | 0 (0) | 0 (0) | 0 (0) | NA |
| **12L (%)** | 2 (0.4) | 2 (0.8) | 0 (0) | 0 (0) | 0.4 |
| **T (%)** | 30 (6.5) | 18 (7.4) | 10 (5.8) | 2 (4.2) | 0.6 |
| **N. of total needle passes (mean ± SD)** | 5.9 (± 2.8) | 5.9 (± 2.9) | 5.5 (± 2.3) | 7.4 (3.2) | 0.0003 |
| **Anesthesiologist assistance (%)** | 56 (12.1) | 2 (0.8) | 7 (4) | 47 (100) | <0.0001 |
| **Sedative drugs** |  |  |  |  |  |
| **Meperidine (mg, mean ± SD)** | 48.5 (± 38.1) | 56.5 (± 36.2) | 50.1 (± 36.3) | 0 (± 0) | <0.0001 |
| **Midazolam (mg, mean ± SD)** | 5.4 (± 2.9) | 5.9 (± 2.7) | 5.5 (± 2.6) | 1.3 (± 2.1) | <0.0001 |
| **Fentanyl (mg, mean ± SD)** | 0.052 (± 0.064) | 0.044 (± 0.059) | 0.047 (± 0.055) | 0.118 (± 0.078) | <0.0001 |
| **Remifentanil (mg, mean ± SD) *** | 0.31 (± 0.9) | 0 (± 0) | 0.36 (± 0.75) | 0.32 (± 1) | 0.64 |
| **Propofol (mg, mean ± SD) *** | 516.8 (± 331.9) | 250 (± 0) | 454.3 (± 296.6) | 550.6 (± 325.8) | 0.13 |
| **Adverse events (%)** | 47 (10.1) | 25 (10.2) | 17 (9.8) | 5 (10.6) | 0.98 |
| **Epistaxis (%)** | 5 (2) | 5 (0) | 0 (0) | 0 (0) | 0.1 |
| **Mild airway bleeding (%)** | 14 (3) | 11 (4.5) | 1 (0.6) | 2 (4.2) | 0.06 |
| **Desaturation (%)** | 8 (1.7) | 1 (0.4) | 6 (3.5) | 1 (2.1) | 0.06 |
| **Hypertension (%)** | 2 (0.4) | 1 (0.4) | 1 (0.6) | 0 (0) | 0.9 |
| **Use of sedative antagonists (%)** | 1 (0.2) | 1 (0.4) | 0 (0) | 0 (0) | 0,6 |
| **Cough (%)** | 6 (1.3) | 2 (0.8) | 4 (2.3) | 0 (0) | 0.3 |
| **Agitation (%)** | 10 (2.2) | 2 (0.8) | 6 (3.4) | 2 (4.2) | 0.1 |
| **Stridor or bronchospasm (%)** | 7 (1.5) | 3 (1.2) | 4 (2.3) | 0 (0) | 0.4 |
| **Diagnostic sample (%)** | 381 (82) | 205 (84) | 142 (82) | 34 (72) | 0.16 |
| **Chronic inflammation (%)** | 10 (2.2) | 6 (2.5) | 3 (1.7) | 1 (2.1) | 0.9 |
| **Anthracosis (%)** | 85 (18.3) | 52 (21.3) | 23 (13.3) | 10 (21.3) | 0.09 |
| **Suspicious for malignancy (%)** | 3 (0.6) | 2 (0.8) | 0 (0) | 1 (2.1) | 0.2 |
| **NSCLC (%)** | 177 (38.1) | 96 (39.3) | 65 (37.6) | 16 (34) | 0.4 |
| **SCLC (%)** | 40 (8.6) | 20 (8.2) | 17 (9.8) | 3 (6.4) | 0.7 |
| **Neuroendocrine large cell tumor (%)** | 1 (0.2) | 0 (0) | 1 (0.6) | 0 (0) | 0.4 |
| **Metastasis of other solid tumors (%)** | 19 (4.1) | 10 (4.1) | 8 (4.6) | 1 (2.1) | 0.7 |
| **Benign neoplasms (%)** | 1 (0.2) | 0 (0) | 1 (0.6) | 0 (0) | 0.4 |
| **Sarcoidosis (%)** | 37 (8) | 16 (6.5) | 21 (12.1) | 0 (0) | 0.01 |
| **Lymphoma (%)** | 10 (2.2) | 5 (2) | 3 (1.7) | 2 (4.2) | 0.6 |
| **Lymphocytes (%)** | 234 (50.4) | 129 (52.9) | 79 (45.7) | 26 (55.3) | 0.3 |
| **Inadequate sample (%)** | 24 (5.2) | 12 (4.9) | 9 (5.2) | 3 (6.4) | 0.9 |
| **False negative (%)** | 2 (0.4) | 2 (0.8) | 0 (0) | 0 (0) | 0.4 |

Table S2. Anagraphics, data regarding samples, procedural sedation, adverse events and results comparison of the nose and mouth access groups. * Mean, SD and p values of propofol and remifentanil have been calculated taking into account only cases with anesthesiologist assistance. SD, standard deviation; BMI, body mass index; NSCLC, non-small cell lung cancer; SCLC, small cell lung cancer.

|  | **Nose (244)** | **Mouth (173)** | **p value** |
| --- | --- | --- | --- |
| **Age (years, mean ± SD)** | 69.1 (± 11.5) | 68.3 (± 11.4) | 0.49 |
| **Sex (males, %)** | 169 (69.3) | 96 (55.5) | 0.005 |
| **Death (%)** | 123 (50.4) | 94 (54.3) | 0.49 |
| **Weight (kg, mean ± SD)** | 74.8 (± 16.4) | 71.7 (± 16.8) | 0.03 |
| **Height (cm, mean ± SD)** | 168.1 (± 10.3) | 165.4 (± 9.2) | 0.005 |
| **BMI (kg/m^2^, mean ± SD)** | 26.3 (± 4.6) | 26 (± 5.4) | 0.25 |
| **Previous biopsies (%)** | 37 (15.2) | 29 (16.8) | 0.68 |
| **Mediastinal staging (%)** | 74 (30.3) | 37 (21.4) | 0.04 |
| **Mediastinal adenopathies (%)** | 231 (94.7) | 168 (97.1) | 0.33 |
| **Adequate visualization (%)** | 238 (98.3) | 170 (97.5) | 0.74 |
| **Sample acquisition (%)** | 240 (98.4) | 171 (98.8) | >0.9 |
| **N. of stations sampled (mean ± SD)** | 1.7 (± 1.1) | 1.6 (± 0.9) | 0.47 |
| **2R (%)** | 7 (2.9) | 0 (0) | 0.045 |
| **2L (%)** | 3 (1.2) | 0 (0) | 0.27 |
| **3 (%)** | 2 (0.8) | 0 (0) | 0.51 |
| **4R (%)** | 93 (38.1) | 62 (35.8) | 0.68 |
| **4L (%)** | 34 (13.9) | 16 (9.2) | 0.17 |
| **7 (%)** | 138 (56.6) | 103 (59.5) | 0.55 |
| **8 (%)** | 2 (0.8) | 1 (0.6) | >0.9 |
| **10R (%)** | 7 (2.9) | 9 (5.2) | 0.3 |
| **10L (%)** | 8 (3.3) | 5 (2.9) | >0.9 |
| **11R (%)** | 57 (23.4) | 36 (20.8) | 0.55 |
| **11L (%)** | 40 (16.4) | 23 (19.1) | 0.51 |
| **12R (%)** | 0 (0) | 0 (0) | >0.9 |
| **12L (%)** | 2 (0.8) | 0 (0) | 0.51 |
| **T (%)** | 18 (7.4) | 10 (5.8) | 0.56 |
| **N. of total needle passes (mean ± SD)** | 5.9 (± 2.9) | 5.5 (± 2.3) | 0.1 |
| **Anesthesiologist assistance (%)** | 2 (0.8) | 7 (4) | 0.037 |
| **Sedative drugs** |  |  |  |
| **Meperidine (mg, mean ± SD)** | 56.5 (± 36.2) | 50.1 (± 36.3) | 0.08 |
| **Midazolam (mg, mean ± SD)** | 5.9 (± 2.7) | 5.5 (± 2.6) | 0.14 |
| **Fentanyl (mg, mean ± SD)** | 0.044 (± 0.059) | 0.047 (± 0.055) | 0.58 |
| **Remifentanil (mg, mean ± SD) *** | 0 (± 0) | 0.36 (± 0.75) | >0.9 |
| **Propofol (mg, mean ± SD) *** | 250 (± 0) | 454.3 (± 296.6) | 0.12 |
| **Adverse events (%)** | 25 (10.2) | 17 (9.8) | >0.9 |
| **Epistaxis (%)** | 5 (0) | 0 (0) | 0.08 |
| **Mild airway bleeding (%)** | 11 (4.5) | 1 (0.6) | 0.017 |
| **Desaturation (%)** | 1 (0.4) | 6 (3.5) | 0.022 |
| **Hypertension (%)** | 1 (0.4) | 1 (0.6) | >0.9 |
| **Use of sedative antagonists (%)** | 1 (0.4) | 0 (0) | >0.9 |
| **Cough (%)** | 2 (0.8) | 4 (2.3) | 0.24 |
| **Agitation (%)** | 2 (0.8) | 6 (3.4) | 0.07 |
| **Stridor or bronchospasm (%)** | 3 (1.2) | 4 (2.3) | 0.45 |
| **Diagnostic sample (%)** | 205 (84) | 142 (82) | 0.59 |
| **Chronic inflammation (%)** | 6 (2.5) | 3 (1.7) | 0.74 |
| **Anthracosis (%)** | 52 (21.3) | 23 (13.3) | 0.039 |
| **Suspicious for malignancy (%)** | 2 (0.8) | 0 (0) | 0.51 |
| **NSCLC (%)** | 96 (39.3) | 65 (37.6) | 0.3 |
| **SCLC (%)** | 20 (8.2) | 17 (9.8) | 0.6 |
| **Neuroendocrine large cell tumor (%)** | 0 (0) | 1 (0.6) | 0.41 |
| **Metastasis of other solid tumors (%)** | 10 (4.1) | 8 (4.6) | 0.81 |
| **Benign neoplasms (%)** | 0 (0) | 1 (0.6) | 0.41 |
| **Sarcoidosis (%)** | 16 (6.5) | 21 (12.1) | 0.055 |
| **Lymphoma (%)** | 5 (2) | 3 (1.7) | >0.9 |
| **Lymphocytes (%)** | 129 (52.9) | 79 (45.7) | 0.16 |
| **Inadequate sample (%)** | 12 (4.9) | 9 (5.2) | >0.9 |
| **False negative (%)** | 2 (0.8) | 0 (0) | 0.51 |

Table S3. Anagraphics, data regarding samples, procedural sedation, adverse events and results comparison of the nose and laryngeal mask (i-Gel) access groups. * Mean, SD and p values of propofol and remifentanil have been calculated taking into account only cases with anesthesiologist assistance. SD, standard deviation; BMI, body mass index; NSCLC, non-small cell lung cancer; SCLC, small cell lung cancer.

|  | **Nose (244)** | **Laryngeal mask (47)** | **p value** |
| --- | --- | --- | --- |
| **Age (years, mean ± SD)** | 69.1 (± 11.5) | 68.1 (± 11.9) | 0.62 |
| **Sex (males, %)** | 169 (69.3) | 31 (66) | 0.73 |
| **Death (%)** | 123 (50.4) | 19 (40.4) | 0.26 |
| **Weight (kg, mean ± SD)** | 74.8 (± 16.4) | 73.3 (± 12.9) | 0.96 |
| **Height (cm, mean ± SD)** | 168.1 (± 10.3) | 167.3 (± 9.1) | 0.66 |
| **BMI (kg/m^2^, mean ± SD)** | 26.3 (± 4.6) | 26 (± 4.5) | 0.73 |
| **Previous biopsies (%)** | 37 (15.2) | 15 (31.9) | 0.01 |
| **Mediastinal staging (%)** | 74 (30.3) | 25 (53.2) | 0.004 |
| **Mediastinal adenopathies (%)** | 231 (94.7) | 45 (95.7) | >0.9 |
| **Adequate visualization (%)** | 238 (98.3) | 47 (100) | 0.59 |
| **Sample acquisition (%)** | 240 (98.4) | 47 (100) | >0.9 |
| **N. of stations sampled (mean ± SD)** | 1.7 (± 1.1) | 2.1 (± 1.3) | 0.027 |
| **2R (%)** | 7 (2.9) | 2 (4.2) | 0.6 |
| **2L (%)** | 3 (1.2) | 0 (0) | >0.9 |
| **3 (%)** | 2 (0.8) | 1 (2.1) | 0.4 |
| **4R (%)** | 93 (38.1) | 18 (38.3) | >0.9 |
| **4L (%)** | 34 (13.9) | 14 (29.8) | 0.02 |
| **7 (%)** | 138 (56.6) | 32 (68.1) | 0.2 |
| **8 (%)** | 2 (0.8) | 0 (0) | >0.9 |
| **10R (%)** | 7 (2.9) | 7 (14.9) | 0.003 |
| **10L (%)** | 8 (3.3) | 1 (2.1) | >0.9 |
| **11R (%)** | 57 (23.4) | 15 (31.9) | 0.27 |
| **11L (%)** | 40 (16.4) | 8 (17) | >0.9 |
| **12R (%)** | 0 (0) | 0 (0) | >0.9 |
| **12L (%)** | 2 (0.8) | 0 (0) | >0.9 |
| **T (%)** | 18 (7.4) | 2 (4.2) | 0.75 |
| **N. of total needle passes (mean ± SD)** | 5.9 (± 2.9) | 7.4 (3.2) | 0.0004 |
| **Anesthesiologist assistance (%)** | 2 (0.8) | 47 (100) | <0.0001 |
| **Sedative drugs** |  |  |  |
| **Meperidine (mg, mean ± SD)** | 56.5 (± 36.2) | 0 (± 0) | <0.0001 |
| **Midazolam (mg, mean ± SD)** | 5.9 (± 2.7) | 1.3 (± 2.1) | <0.0001 |
| **Fentanyl (mg, mean ± SD)** | 0.044 (± 0.059) | 0.118 (± 0.078) | <0.0001 |
| **Remifentanil (mg, mean ± SD) *** | 0 (± 0) | 0.32 (± 1) | >0.9 |
| **Propofol (mg, mean ± SD) *** | 250 (± 0) | 550.6 (± 325.8) | 0.06 |
| **Adverse events (%)** | 25 (10.2) | 5 (10.6) | >0.9 |
| **Epistaxis (%)** | 5 (2) | 0 (0) | >0.9 |
| **Mild airway bleeding (%)** | 11 (4.5) | 2 (4.2) | >0.9 |
| **Desaturation (%)** | 1 (0.4) | 1 (2.1) | 0.3 |
| **Hypertension (%)** | 1 (0.4) | 0 (0) | >0.9 |
| **Use of sedative antagonists (%)** | 1 (0.4) | 0 (0) | >0.9 |
| **Cough (%)** | 2 (0.8) | 0 (0) | >0.9 |
| **Agitation (%)** | 2 (0.8) | 2 (4.2) | 0.12 |
| **Stridor or bronchospasm (%)** | 3 (1.2) | 0 (0) | >0.9 |
| **Diagnostic sample (%)** | 205 (84) | 34 (72) | 0.06 |
| **Chronic inflammation (%)** | 6 (2.5) | 1 (2.1) | >0.9 |
| **Anthracosis (%)** | 52 (21.3) | 10 (21.3) | >0.9 |
| **Suspicious for malignancy (%)** | 2 (0.8) | 1 (2.1) | 0.41 |
| **NSCLC (%)** | 96 (39.3) | 16 (34) | 0.33 |
| **SCLC (%)** | 20 (8.2) | 3 (6.4) | >0.9 |
| **Neuroendocrine large cell tumor (%)** | 0 (0) | 0 (0) | >0.9 |
| **Metastasis of other solid tumors (%)** | 10 (4.1) | 1 (2.1) | >0.9 |
| **Benign neoplasms (%)** | 0 (0) | 0 (0) | >0.9 |
| **Sarcoidosis (%)** | 16 (6.5) | 0 (0) | 0.08 |
| **Lymphoma (%)** | 5 (2) | 2 (4.2) | 0.31 |
| **Lymphocytes (%)** | 129 (52.9) | 26 (55.3) | 0.87 |
| **Inadequate sample (%)** | 12 (4.9) | 3 (6.4) | 0.71 |
| **False negative (%)** | 2 (0.8) | 0 (0) | >0.9 |

Table S4. Anagraphics, data regarding samples, procedural sedation, adverse events and results comparison of the mouth and laryngeal mask (i-Gel) access groups. * Mean, SD and p values of propofol and remifentanil have been calculated taking into account only cases with anesthesiologist assistance. SD, standard deviation; BMI, body mass index; NSCLC, non-small cell lung cancer; SCLC, small cell lung cancer.

|  | **Mouth (173)** | **Laryngeal mask (47)** | **p value** |
| --- | --- | --- | --- |
| **Age (years, mean ± SD)** | 68.3 (± 11.4) | 68.1 (± 11.9) | 0.93 |
| **Sex (males, %)** | 96 (55.5) | 31 (66) | 0.24 |
| **Death (%)** | 94 (54.3) | 19 (40.4) | 0.10 |
| **Weight (kg, mean ± SD)** | 71.7 (± 16.8) | 73.3 (± 12.9) | 0.20 |
| **Height (cm, mean ± SD)** | 165.4 (± 9.2) | 167.3 (± 9.1) | 0.25 |
| **BMI (kg/m^2^, mean ± SD)** | 26 (± 5.4) | 26 (± 4.5) | 0.76 |
| **Previous biopsies (%)** | 29 (16.8) | 15 (31.9) | 0.04 |
| **Mediastinal staging (%)** | 37 (21.4) | 25 (53.2) | <0.0001 |
| **Mediastinal adenopathies (%)** | 168 (97.1) | 45 (95.7) | 0.64 |
| **Adequate visualization (%)** | 170 (97.5) | 47 (100) | >0.9 |
| **Sample acquisition (%)** | 171 (98.8) | 47 (100) | >0.9 |
| **N. of stations sampled (mean ± SD)** | 1.6 (± 0.9) | 2.1 (± 1.3) | 0.01 |
| **2R (%)** | 0 (0) | 2 (4.2) | 0.04 |
| **2L (%)** | 0 (0) | 0 (0) | >0.9 |
| **3 (%)** | 0 (0) | 1 (2.1) | 0.21 |
| **4R (%)** | 62 (35.8) | 18 (38.3) | 0.86 |
| **4L (%)** | 16 (9.2) | 14 (29.8) | 0.001 |
| **7 (%)** | 103 (59.5) | 32 (68.1) | 0.40 |
| **8 (%)** | 1 (0.6) | 0 (0) | >0.9 |
| **10R (%)** | 9 (5.2) | 7 (14.9) | 0.05 |
| **10L (%)** | 5 (2.9) | 1 (2.1) | >0.9 |
| **11R (%)** | 36 (20.8) | 15 (31.9) | 0.12 |
| **11L (%)** | 23 (19.1) | 8 (17) | 0.83 |
| **12R (%)** | 0 (0) | 0 (0) | >0.9 |
| **12L (%)** | 0 (0) | 0 (0) | >0.9 |
| **T (%)** | 10 (5.8) | 2 (4.2) | >0.9 |
| **N. of total needle passes (mean ± SD)** | 5.5 (± 2.3) | 7.4 (3.2) | <0.0001 |
| **Anesthesiologist assistance (%)** | 7 (4) | 47 (100) | <0.0001 |
| **Sedative drugs** |  |  |  |
| **Meperidine (mg, mean ± SD)** | 50.1 (± 36.3) | 0 (± 0) | <0.0001 |
| **Midazolam (mg, mean ± SD)** | 5.5 (± 2.6) | 1.3 (± 2.1) | <0.0001 |
| **Fentanyl (mg, mean ± SD)** | 0.047 (± 0.055) | 0.118 (± 0.078) | <0.0001 |
| **Remifentanil (mg, mean ± SD) *** | 0.36 (± 0.75) | 0.32 (± 1) | 0.91 |
| **Propofol (mg, mean ± SD) *** | 454.3 (± 296.6) | 550.6 (± 325.8) | 0.32 |
| **Adverse events (%)** | 17 (9.8) | 5 (10.6) | 0.79 |
| **Epistaxis (%)** | 0 (0) | 0 (0) | >0.9 |
| **Mild airway bleeding (%)** | 1 (0.6) | 2 (4.2) | 0.11 |
| **Desaturation (%)** | 6 (3.5) | 1 (2.1) | >0.9 |
| **Hypertension (%)** | 1 (0.6) | 0 (0) | >0.9 |
| **Use of sedative antagonists (%)** | 0 (0) | 0 (0) | >0.9 |
| **Cough (%)** | 4 (2.3) | 0 (0) | 0.58 |
| **Agitation (%)** | 6 (3.4) | 2 (4.2) | 0.68 |
| **Stridor or bronchospasm (%)** | 4 (2.3) | 0 (0) | 0.58 |
| **Diagnostic sample (%)** | 142 | 34 (72) | 0.15 |
| **Chronic inflammation (%)** | 3 (1.7) | 1 (2.1) | >0.9 |
| **Anthracosis (%)** | 23 (13.3) | 10 (21.3) | 0.17 |
| **Suspicious for malignancy (%)** | 0 (0) | 1 (2.1) | 0.21 |
| **NSCLC (%)** | 65 (37.6) | 16 (34) | 0.73 |
| **SCLC (%)** | 17 (9.8) | 3 (6.4) | 0.59 |
| **Neuroendocrine large cell tumor (%)** | 1 (0.6) | 0 (0) | >0.9 |
| **Metastasis of other solid tumors (%)** | 8 (4.6) | 1 (2.1) | 0.69 |
| **Benign neoplasms (%)** | 1 (0.6) | 0 (0) | >0.9 |
| **Sarcoidosis (%)** | 21 (12.1) | 0 (0) | 0.009 |
| **Lymphoma (%)** | 3 (1.7) | 2 (4.2) | 0.29 |
| **Lymphocytes (%)** | 79 (45.7) | 26 (55.3) | 0.25 |
| **Inadequate sample (%)** | 9 (5.2) | 3 (6.4) | 0.72 |
| **False negative (%)** | 0 (0) | 0 (0) | >0.9 |
